# The effects of acacia honey on in vitro corneal abrasion wound healing model

**DOI:** 10.1186/s12860-015-0053-9

**Published:** 2015-02-18

**Authors:** Choy Ker-Woon, Norzana Abd Ghafar, Chua Kien Hui, Yasmin Anum Mohd Yusof, Wan Zurinah Wan Ngah

**Affiliations:** Department of Anatomy, Level 18, Pre-Clinical Block, Universiti Kebangsaan Malaysia Medical Centre (UKMMC), Jalan Yaacob Latif, Bandar Tun Razak, Cheras, 56000 Kuala Lumpur, Malaysia; UKM Medical Molecular Biology Institute (UMBI), Jalan Yaacob Latif, Bandar Tun Razak, Cheras, 56000 Kuala Lumpur, Malaysia; Department of Physiology, Universiti Kebangsaan Malaysia Medical Centre (UKMMC), Jalan Yaacob Latif, Bandar Tun Razak, Cheras, 56000 Kuala Lumpur, Malaysia; Department of Biochemistry, Universiti Kebangsaan Malaysia Medical Centre (UKMMC), Jalan Yaacob Latif, Bandar Tun Razak, Cheras, 56000 Kuala Lumpur, Malaysia

**Keywords:** Acacia honey, Corneal epithelial cells, Migration, Corneal abrasion, Corneal wound healing

## Abstract

**Background:**

Acacia honey (AH) has been proven to improve skin wound healing, but its therapeutic effects on corneal epithelium has not been elucidated to date. This study aimed to investigate the effects of AH on cultured corneal epithelial cells (CEC) on in vitro corneal abrasion wound healing model. Six New Zealand white rabbits’ CEC were isolated and cultured until passage 1. Circular wound area was created onto a confluent monolayer CEC using a corneal trephine which mimicked corneal abrasion and treated with 0.025% AH supplemented in basal medium (BM) and complete cornea medium (CCM). Wound healing was measured as the percentage of wound closure by the migration of CEC on day 0, day 3 and day 6, post wound creation. The morphological changes of CEC were assessed via phase contrast microscopy. Gene and protein expressions of cytokeratin (CK3), fibronectin and cluster of differentiation 44 (CD44) in AH treated groups and control groups were determined by real-time PCR and immunocytochemistry, respectively.

**Results:**

Cultured CEC exhibited similar morphology of polygonal shaped cells in all culture media. CEC cultured in AH-supplemented media showed higher percentage of wound closure compared to the controls. Gene expression of CK3 increased in AH-supplemented groups throughout the study. Fibronectin expression was increased at the initial stage while CD44 expression was increased at day 3, post wound creation. The protein expression of CEC cultured in all media was in accordance to their respective gene expressions.

**Conclusion:**

Supplementation of AH in BM and CCM media accelerates CEC wound closure of the in vitro corneal abrasion model by increasing the expression of genes and proteins associated with CEC wound healing.

## Background

Cornea is composed of five separate layers i.e. an outer stratified layer of squamous non-keratinized epithelium, Bowman’s membrane, stroma layer, Descemet’s membrane and the innermost endothelial layer. Corneal epithelium serves as the first line barrier against infections or insults from harmful environmental agents. In superficial corneal injury such as corneal abrasion which is often caused by mechanical injuries such as trauma or chemical burn [1], the corneal epithelial integrity is affected [2]. Thus, a faster rate of wound healing of the corneal epithelium is vital for optimal function and protection of the inner structures of the cornea. Corneal epithelial wound healing occurs in three phases i.e. migration, cell proliferation, and remodelling [3].

Migration and proliferation of epithelial cells are essential for wound closure during initial phase of corneal epithelial wound healing. Epithelial cells express the specific corneal epithelial differentiation marker, cytokeratin 3 (CK3) and cytokeratin 12 (CK12) once the cells left the limbal basal layer during centripetal migration [4]. These cells also expressed fibronectin, a prototype of cell adhesion protein required for cell attachment, migration, differentiation, wound healing and cytoskeletal organization [5,6]. The migration phase in cornea wound healing is influenced by the synthesis of cluster of differentiation 44 (CD44), the primary cell surface receptor for hyaluronate (HA) receptor [7].

The standard treatment for corneal abrasion is application of topical antibiotic or antifungal eye drop in order to prevent secondary infection. However, these antibiotics can cause microbial resistance in the long run [8] while preservative such as benzalkonium ammonium chloride (BAK) in eye drops may result in disruption of corneal epithelium, thereby delaying wound healing [9].

Honey has been documented to have wound healing properties on skin as it possesses antibacterial, anti-inflammatory and antioxidant functions [10-12]. It has high sugar content flavonoids, phenolic acids, organic acids and mineral in different compositions depending on its floral and geographical source [13]. Since cornea and skin are both derived from surface ectoderm embryologically, we hypothesized that honey could have the same potential effects in accelerating the migration and proliferation of CEC during corneal epithelial wound healing.

Acacia honey (AH) is a local honey in Malaysia collected by *Apis mellifera* honeybees from Acacia mangium trees [14]. AH was reported to promote wound contraction resulting from burn injury [15] but its therapeutic effects on corneal epithelium still remains unknown. In the present study, we have established an in vitro corneal abrasion wound healing model aiming for quantitative evaluation of the effects of AH on the migration and healing properties of CEC during wound healing.

## Methods

This study was conducted following approval obtained from the Research and Ethical Committee, Faculty of Medicine, Universiti Kebangsaan Malaysia (UKM project code: GGPM-2011-085) and Universiti Kebangsaan Malaysia Animal Ethics Committee (project code: UKMAEC Approval Number FP/ANAT/2012/NORZANA/18-JANUARY/419-JANUARY-2011-DECEMBER-2013-AR-CAT2).

### Acacia honey (AH)

Acacia honey (AH) was purchased from Ministry of Agriculture, Malaysia and gamma irradiated at 25 kGy at Ministry of Science, Technology and Innovation, Malaysia. The optimal concentration of AH was identified as 0.025% according to our previous study [16].

### Rabbit corneal epithelial cells isolation and culture

CEC from six New Zealand White strain rabbits’ corneas were removed, isolated and culture expanded as described previously [17]. In brief, the corneas were cut 2 mm beyond the cornea-scleral junction. The unwanted connective tissue such as ocular muscles, iris and conjunctiva were removed. The endothelium was gently scraped off. The corneas were rinsed with phosphate buffered solution (Gibco Invitrogen, USA) before incubation in Dispase solution 2 mg/ml (Sigma-Aldrich, USA) at 4°C for 18 hours to separate the epithelium from the stroma. Using a fine surgical blade, the epithelial layer was gently removed followed by digestion with 5 ml of 0.05% trypsin-EDTA (Gibco Invitrogen, USA) in a centrifuge tube to obtain single cell suspension. Five ml of define trypsin inhibitor (Gibco Invitrogen, USA) were added to neutralize the reaction of trypsin-EDTA and was centrifuged at 500 × g for 10 minutes. The resultant pellet was suspended in complete cornea medium (CCM) containing human corneal growth supplement (HCGS) and antibiotic antimycotic (Gibco, Invitrogen, USA). Viable CEC were seeded in six well-plates (BD Falcon, Franklin Lakes, NJ) with seeding density of 1 × 10^5^ cells per well. Cells were cultured in 5% CO2 incubator (Jouan, Duguay Trouin, SH) under 95% humidity at 37°C. Upon 80% confluence, cells were trypsinized with 1 ml of versene (Gibco, Invitrogen, USA) and 0.05% trypsin-EDTA and subcultured until passage 1 (P1). Media were changed every 2 days. The CEC morphological changes were examined everyday with inverted phase contrast microscope (Carl Zeiss, Germany).

### *In vitro* corneal abrasion wound healing model

**Figure 1:**
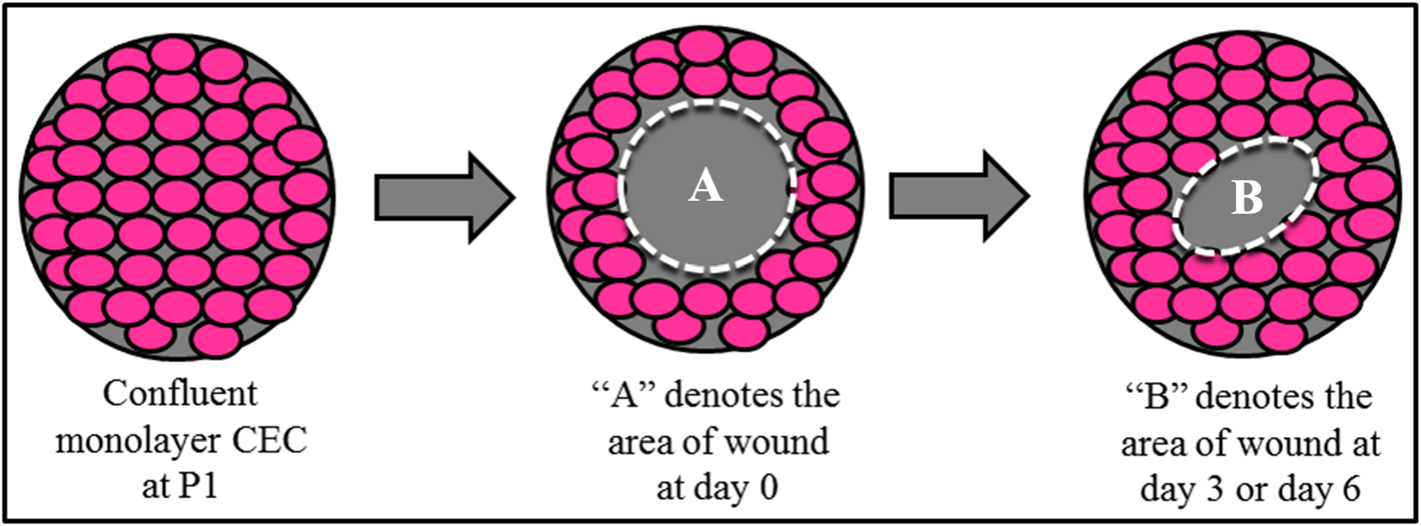
**Schematic diagram of in vitro corneal abrasion model onto confluent monolayer CEC at P1.** The stages are shown with arrows.

CEC were seeded in six well-plates (BD Falcon, Franklin Lakes, NJ) with seeding density of 1 × 10^5^ cells per well and cultured in CCM medium until confluence at day 3. A 4 mm corneal trephine was used to create a circular defect onto the confluent monolayer CEC which was devoid of cells at the centre (Figure 1). The CEC culture were then treated with 4 different media; A) basal medium (BM), B) BM with supplementation of 0.025% AH, C) complete cornea medium (CCM) and D) CCM with supplementation of 0.025% AH. The cultures were maintained at 37°C in a 5% CO_2_ incubator with 95% humidity. The wound area was measured using Axiovision LE64 software on initial day of wounding (day 0), day 3 and day 6 post wound creation (Figure 1). The percentage of wound healing was calculated using the formula below. Formula:

$$ \mathrm{X}=\frac{\mathrm{A}\hbox{-} \mathrm{B}}{\mathrm{A}}\times \kern0.5em 100\% $$

X = Percentage of wound healing.

A = Initial area of wounding.

B = Area of wounding at day 3 or day 6.

### Quantitative real time-PCR (qRT-PCR) for evaluation of corneal wound healing markers

Total RNA from CEC cultured in 4 different media (BM and CCM with and without supplementation of 0.025% AH) at day 0, day 3 and day 6 post in vitro wound creation were extracted using TRI Reagent (Molecular Research Centre, Cincinnati, USA). The lysate was centrifuged at 12000 rpm at 4°C for 5 minutes with Jouan centrifuge (Jouan, Duguay Trouin, SH). Chloroform was added and mixed vigorously for 10 seconds. The mixture was centrifuged at 12000 rpm for 15 minutes at 4°C to isolate the transparent aqueous contained total RNA. Isopropanol and polyacryl carrier (Molecular Research Centre, USA) was added to each extraction to precipitate the total RNA. The extracted RNA pellet was rinsed with 75% ethanol and air dried. Rnase and Dnase free distilled water (Invitrogen, Carlsbad, USA) were then added to dissolve the RNA pellet. Following that, complementary DNA was produced from 100 ng of Total RNA with SuperScript™ III First-Strand Synthesis SuperMix reverse transcriptase (Invitrogen, Carlsbad, USA) using manufacturer’s protocol. In brief, the protocol was 10 minutes at 23°C for primer annealing, 60 minutes at 50°C for reverse transcription and 5 minutes at 85°C for reaction termination. Real-time polymerase chain reaction (Invitrogen, Carlsbad, USA) was performed using SYBR Green as indicator in Bio-Rad iCycler (Bio-Rad, USA). RT-PCR was done using a mixture of 12.5 μl of iQ SYBR Supermix, 1 μl of forward and reverse primers, deionised water and 1 μl of cDNA template. The primers were designed based on NIH GenBank database using Primer-3 (http://primer3.ut.ee/). The reaction profile for all primer pairs was: cycle 1: 95°C for 3 minutes (1 ×), cycle 2: Step 1 for 95°C in 10 seconds and Step 2 for 61°C in 30 seconds (40 ×), followed by melting curve analysis. The corneal wound healing markers evaluated were cytokeratin 3 (CK3), fibronectin and cluster of differentiation 44 (CD44) with GAPDH as internal control. The description of primers used for qRT-PCR is shown in Table 1. The specificity and product size of each PCR product were visualised by 2% agarose gel electrophoresis containing ethidium bromide staining.

**Table 1:** **Description of primers used for qRT-PCR**

**Primer**	**Accession number**	**Primer sequence**	**Product size**
GAPDH	NM_001082253	F:caa cga att tgg cta cag ca	186
		R:aaa ctg tga aga ggg gca ga	
CK3	XM_002711005	F:gac tcg gag ctg aga agc at	198
		R:cag ggt cct cag gaa gtt ga	
Fibronectin	XM_002712573	F:gga atg cac cag aac cat ct	201
		R:agt cga agc gtg tca cct ct	
CD44	XM_002709049	F:cat cct cac ctc caa cac ct	202
		R:gtt gct ggg att gat gtc ct	

### Immunocytochemistry

CEC of the corneal abrasion wound model were fixed in 4% paraformaldehyde at 4°C overnight. Immunocytochemistry was performed using standard protocol from Dako Animal Research Kit. Cultures were washed with running tap water for 3 minutes prior to incubation with blocking agent; 0.03% peroxidase block, at room temperature for 5 minutes. Specimens were labelled using the Biotinylation reagent and primary antibodies were added for incubation. Primary antibodies used were anti-CK3 (1:200, Dako) and anti-fibronectin (1:200, Dako). After 30 minutes, biotinylation reagent was added to bind biotinylated secondary antibody to the primary antibody. The specimens were then incubated with streptavidin-peroxidase and followed by substrate-chromogen. Nuclei were counterstained with haematoxylin (Sigma Aldrich Co, USA) and the culture slides were mounted using DPX mounting medium (Sigma Aldrich Co, USA). The slides were observed using confocal laser scanning microscopy (LSM-510, Zeiss). Positive stained cells showed brownish precipitates in the cytoplasm and the nuclei were stained blue.

### Statistical analysis

Results were expressed as mean ± standard error of mean (SEM). The data were evaluated with paired t-test and ANOVA using SPSS version 20.0 and p <0.05 was considered to be significant.

## Results

### Morphological evaluation

**Figure 2:**
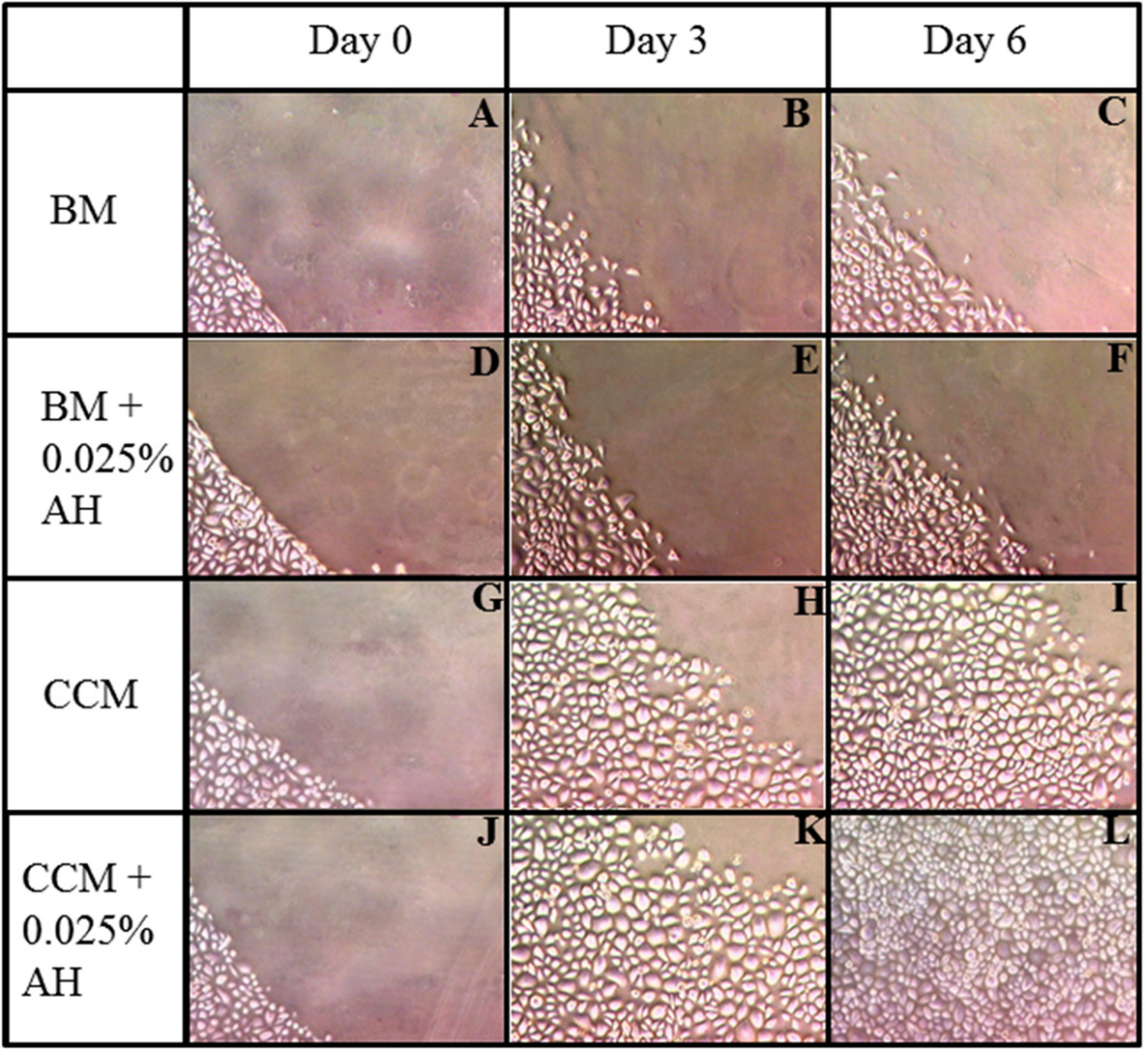
**Phase contrast micrograph showed one ninth of the wound margin of CEC cultured in 4 different media; 1) BM medium (A, B, C), 2) BM supplemented with 0.025% AH (D, E, F), 3) CCM medium (G, H, I), and 4) CCM supplemented with 0.025% AH (J, K, L).** The healing of CEC was evaluated at day 0, day 3 and day 6 post wounding (Magnification × 50).

CEC supplemented with AH exhibited small polygonal-shaped cells with distinct cell border (Figure 2A-L). This indicates supplementation of AH into culture media did not change the morphology of the cultured CEC. CEC appeared large and possessed higher cell density at the wound edge especially when cultured in AH-supplemented media (Figure 2L).

### Corneal epithelial wound healing study

**Figure 3:**
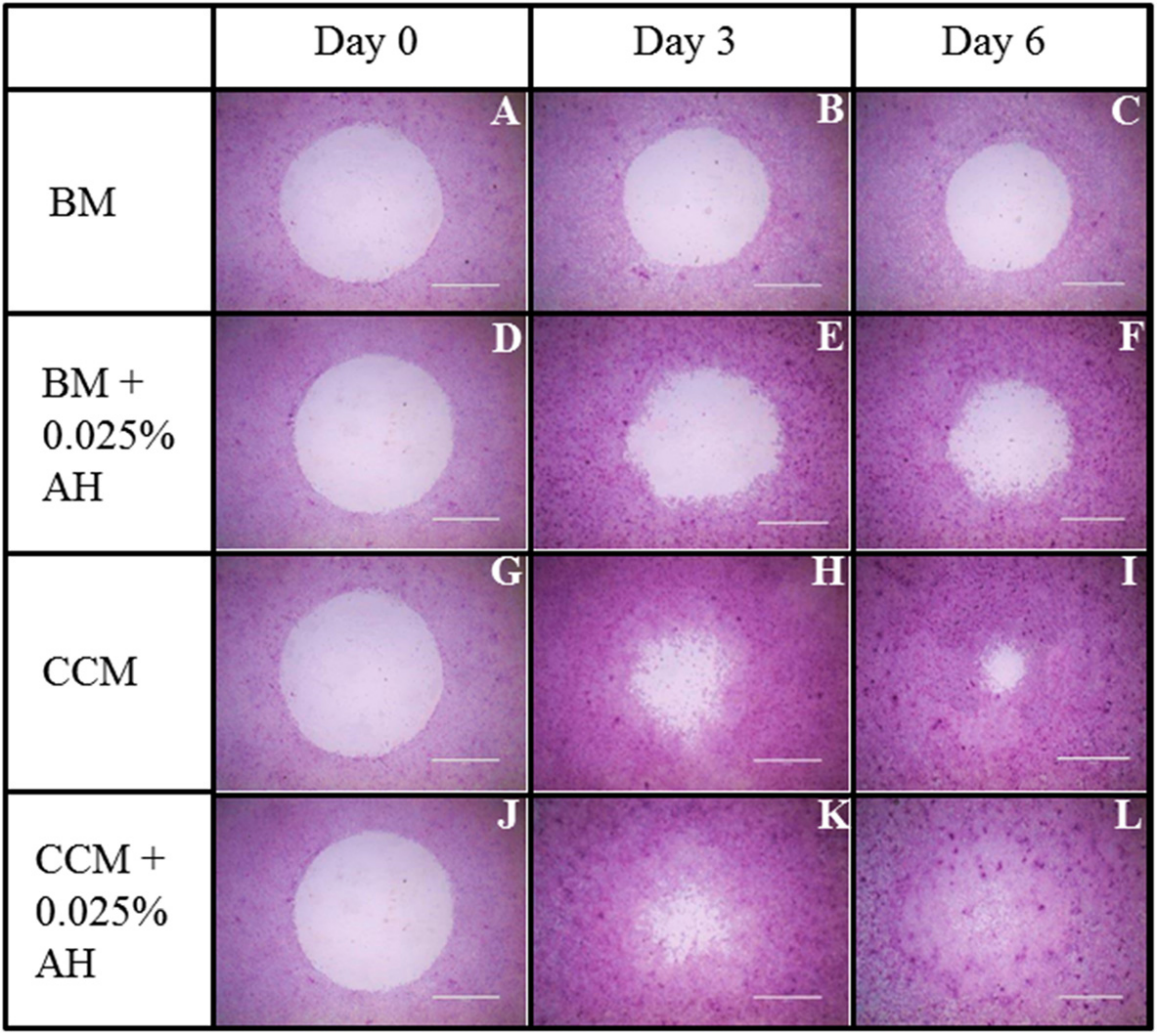
**Micrograph showed the wound area stained with H & E of the cultured CEC in 4 different media; 1) BM medium (A, B, C), 2) BM supplemented with 0.025% AH (D, E, F), 3) CCM medium (G, H, I), and 4) CCM supplemented with 0.025% AH (J, K, L).** The healing of CEC was evaluated at day 0, day 3 and day 6 post wounding (Magnification × 20).

**Figure 4:**
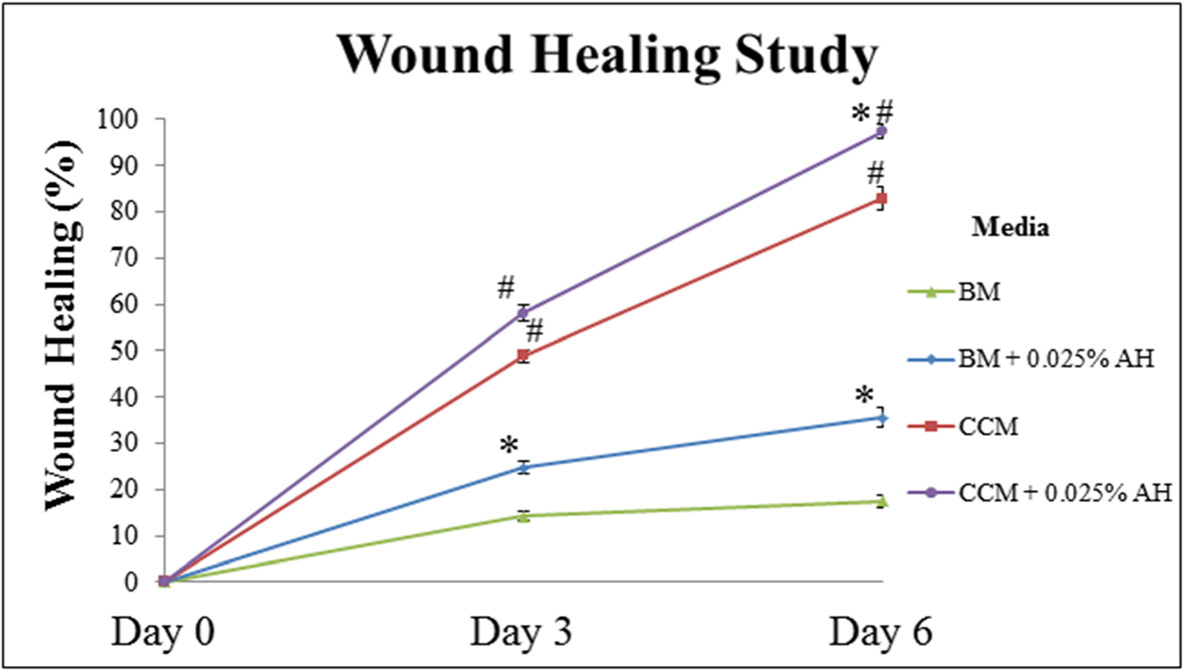
**Line chart showed wound healing study of CEC of in vitro corneal abrasion model cultured in 4 different media (n = 6 independent experiments).** (*) Denotes significant difference (p < 0.05) in the same media with or without AH supplementation (BM compared to BM + 0.025% AH, CCM compared to CCM + 0.025% AH). (#) Denotes significant difference (p < 0.05) between media with or without AH supplementation (BM compared to CCM, BM + 0.025% AH compared to CCM + 0.025% AH).

CEC cultured in AH-supplemented media (Figure 3D-F, J-L) showed faster wound healing compared to the controls (Figure 3A-C, G-I). On day 3 of post wound creation, CEC cultured in BM medium with and without supplementation of AH showed 24.7% and 14.3% wound closure, respectively (Figure 4). CEC cultured in AH-supplemented CCM medium showed 58.0% of wound closure compared to 48.9% for CEC cultured in CCM alone.

At day 6, CEC cultured in AH-supplemented BM media showed 35.6% of the wound closure compared to only 17.4% for CEC cultured in BM medium alone. CEC cultured in CCM media migrated centripetally and wound closure was 82.8%. Wound closed completely at day 6 for CEC cultured in CCM supplemented with AH (Figure 3L).

### Gene expression analysis

**Figure 5:**
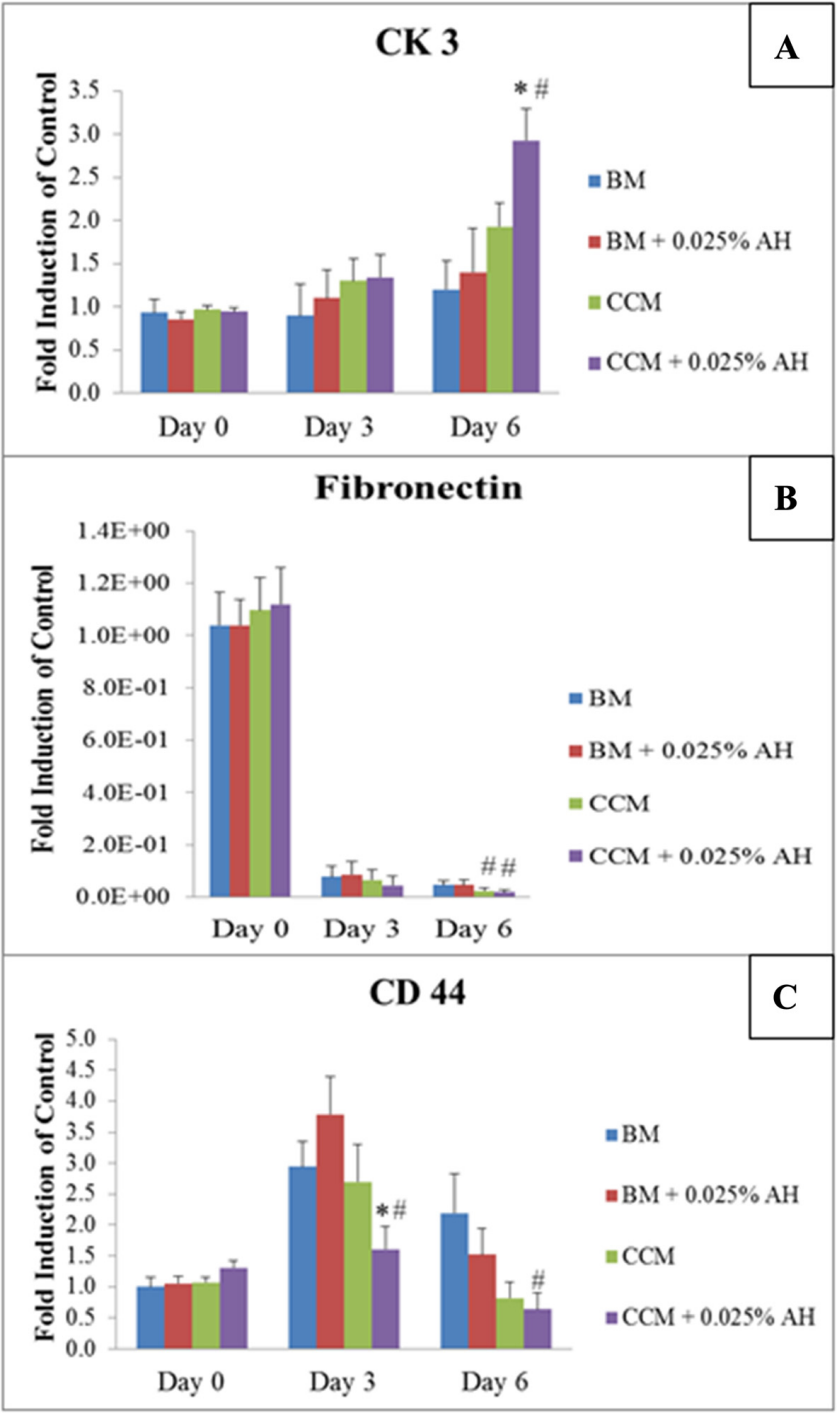
**Gene expression of corneal wound healing markers; (A) CK3, (B) Fibronectin and (C) CD44 for CEC cultured in 4 different media for 6 independent experiments.** Values were expressed as mean ± SEM, n = 6. (*) Denotes significant difference (p < 0.05) in the same media with or without AH supplementation (BM compared to BM + 0.025% AH, CCM compared to CCM + 0.025% AH). (#) Denotes significant difference (p < 0.05) between media with or without AH supplementation (BM compared to CCM, BM + 0.025% AH compared to CCM + 0.025% AH).

The expression of CK3 gene was increased by 0.5 fold in CEC cultured in AH-supplemented BM medium while CEC cultured in AH-supplemented CCM medium showed the highest expression (three fold increment) at day 6 compared to control (Figure 5A).

Gene expression of fibronectin was the highest at the initial day of wound creation (Figure 5B). However, the expression was reduced by ten folds at day 3 and day 6 in all culture media. The expression of fibronectin was significantly reduced in the CCM groups compared to the BM groups at day 6 of the experiment.

Compared to the initial day of wound creation, CD44 gene expression was increased by three folds at day 3 for CEC cultured in BM and CCM groups while for CEC cultured in AH-enriched BM and AH-enriched CCM media, the expression of CD44 was increased by 3.75 and 1.5 folds respectively. The gene expression of CD44 was the lowest in the AH-enriched CCM medium at day 6.

**Figure 6:**
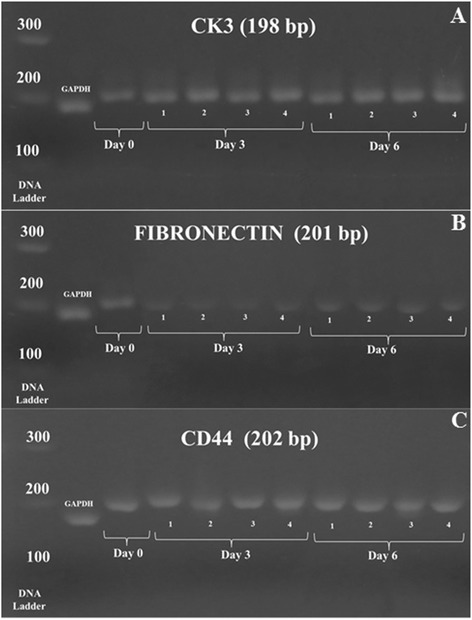
**Gel electrophoresis of (A) CK 3, (B) Fibronectin, (C) CD 44 for the confirmation of product size of cultured CEC in 4 different media, lane 1: BM, lane 2: BM supplemented with 0.025% AH, lane 3: CCM and lane 4: CCM supplemented with 0.025% AH.**

Gel electrophoresis showed the presence of single band indicating specific size product for CK3, fibronectin and CD44 in all culture media (Figure 6).

### Immunocytochemistry

**Figure 7:**
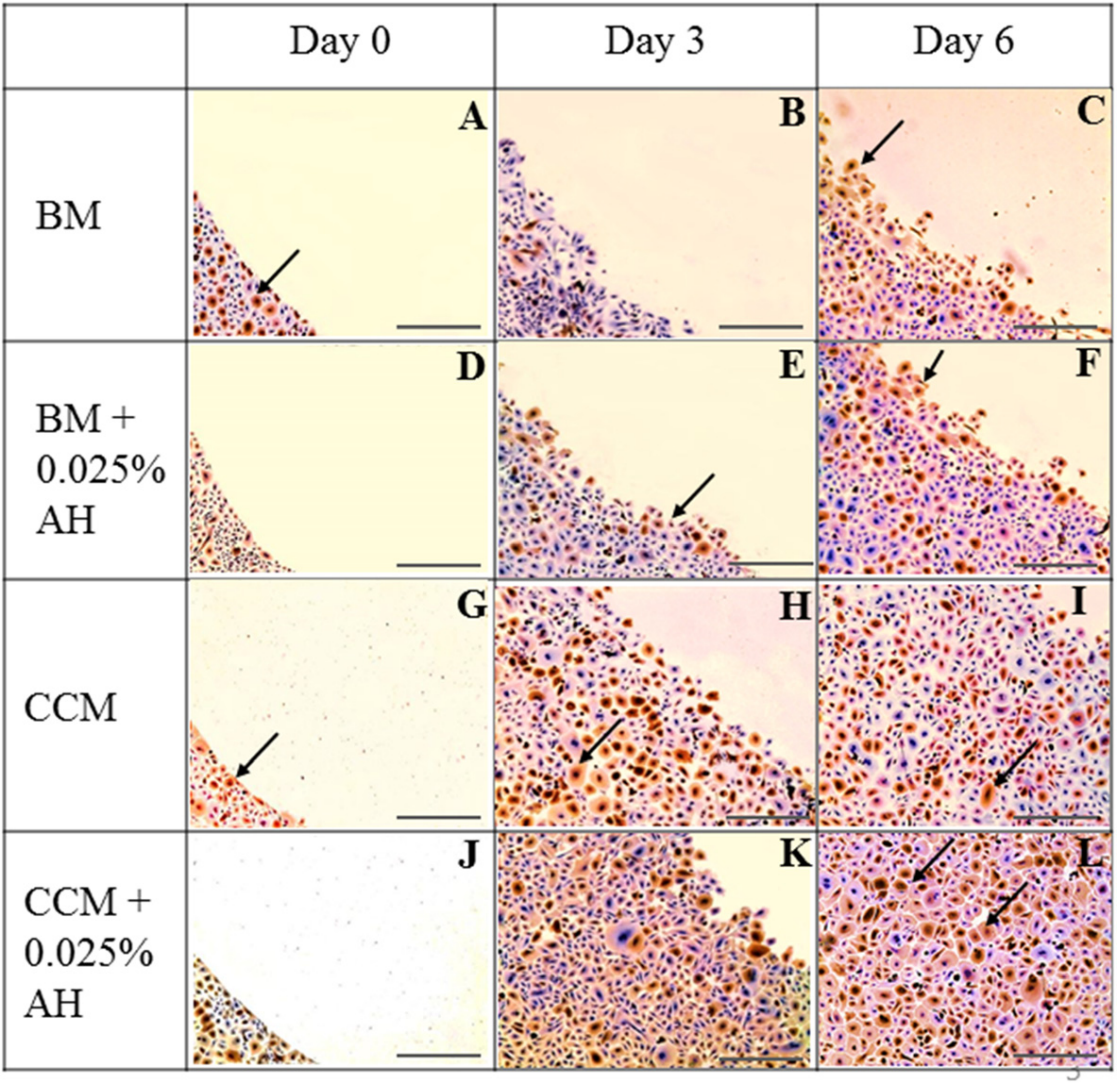
**Immunocytochemistry for CK3 protein expression of CEC cultured in 4 different media; 1) BM medium (A, B, C), 2) BM supplemented with 0.025% AH (D, E, F), 3) CCM medium (G, H, I), and 4) CCM supplemented with 0.025% AH (J, K, L) at day 0, day 3 and day 6.** Positive stained cells are marked with arrows. (Magnification × 50).

Immunocytochemistry for CK3 protein was detected in CEC cultured in all media throughout the study period. CEC cultured in AH-enriched media showed increased immunodeposition of CK3 compared to controls which were in conformity with the gene expression in qRT-PCR analyses (Figure 7).

**Figure 8:**
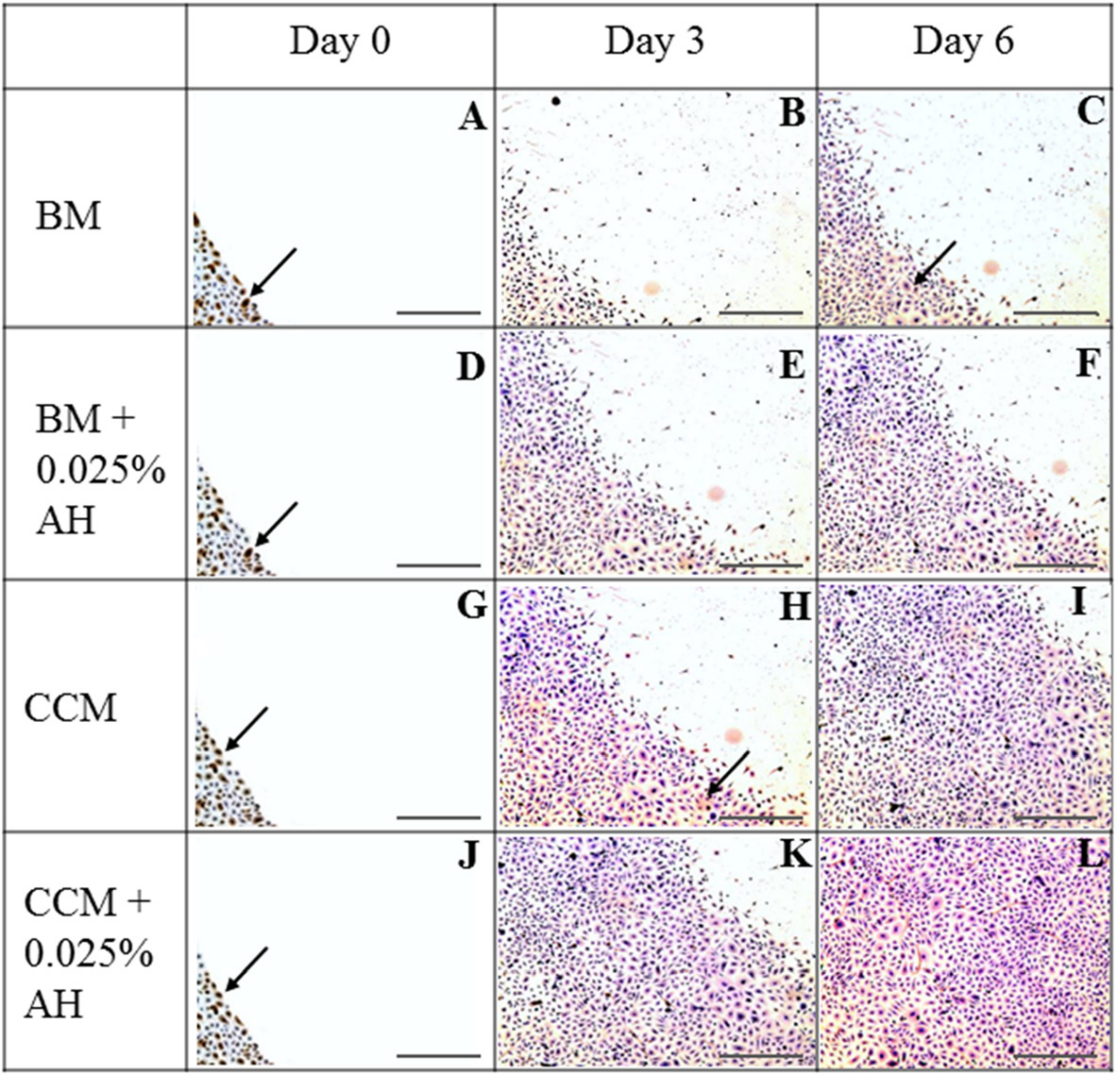
**Immunocytochemistry for Fibronectin protein expression of CEC cultured in 4 different media; 1) BM medium (A, B, C), 2) BM supplemented with 0.025% AH (D, E, F), 3) CCM medium (G, H, I), and 4) CCM supplemented with 0.025% AH (J, K, L) at day 0, day 3 and day 6.** Positive stained cells are marked with arrows. (Magnification × 50).

Fibronectin protein was detected at wound area in all media during initial day of wound creation (Figure 8). However, fibronectin expression was not apparently detected in CEC cultured in all media at day 3 and day 6 which was in agreement with gene expression results.

## Discussion

Migration of the corneal epithelial cells following injury occurs in two ways. Daughter cells of the epithelium called transient amplifying cells (TAC) migrates centripetally with convex leading border into the basal layer of the corneal epithelium and differentiate into the upper layers of the corneal epithelium to become post-mitotic cells. Simultaneously, the epithelial cells undergo preferential circumferential migration which encircles the limbus until the migrating cells meet together [18,19]. Using our in vitro corneal abrasion model, the CEC was found to migrate centripetally following 24 hours post wound creation. An earlier in vivo study reported the migration of epithelium from limbus to the centre of the cornea took 48 to 72 hours following chemical injury [20].

The migration phase in the corneal epithelium are greatly metabolic and depends on the glucose provided in the aqueous humor and epithelial glycogen stores as the main source of energy [21]. During wounding, glucose transporter protein 1 (GLUT1) expression was elevated in the corneal epithelial cell membranes and limbal basal to facilitate and transport glucose to the glucose-starved epithelium [4]. It was documented that the expression of GLUT1 doubled at wound area following 4 hours of injury and continued to rise even after epithelial wound closure [22]. In the present study, we showed the migration of CEC was accelerated in the AH-supplemented media. Glucose, the main composition in honey is responsible for energy production through glycolysis [23]. AH possesses the highest total sugar contents (68.40%) compared to any other local honey [14]. Glucose in AH may have provided additional energy resources via GLUT1 accelerating the migration of CEC for wound closure in the corneal abrasion in vitro model.

Honey efficiently produces a slow-release supply of hydrogen peroxide, a component in honey activated by the enzyme glucose oxidase when honey is diluted [24]. Hydrogen peroxide in honey is well known for its antibacterial property. Bacterial infection is also known to delay wound healing [12,25]. The activation of hydrogen peroxide in AH-supplemented media serves as an ideal culture condition for CEC, thus accelerating the migration of CEC in closing the wounded area. Previous study reported that hydrogen peroxide in low concentration was found to assist wound healing by stimulating epithelial cells migration from wound edges and promoting fibroblasts growth during inflammatory stage [26]. During in vivo wound healing, hydrogen peroxide was reported to provide sufficient oxygen and nutrient to the healing tissues via angiogenesis [27].

AH is known to possess the highest total contents of trace elements, i.e. aluminium (Al), chromium (Cr), caesium (Cs), copper (Cu)*,* ferum (Fe), indium (In), potassium (K), magnesium (Mg), manganese (Mn), sodium (Na), rubidium (Rb), strontium (Sr), uranium (U) and zinc (Zn) compared to other local honey [28]. It has been documented that Zn accelerates wound contraction in full-thickness incision wound during the initial stage of wound healing [29], which further support the results from the present study.

CK3 is a basic keratin pair of CK12 and its expression is an indication of terminally differentiated epithelial cells [29]. Higher expression of CK3 gene in CEC cultured in AH-supplemented media proved that AH promotes centripetal migration of CEC for wound closure. These findings were in agreement with the cell migration study. The expression of CK3 was higher in CCM group which contains human corneal growth factors (HCGS), epidermal growth factor (EGF) and bovine pituitary extract (BPE). EGF has been documented to stimulate proliferation, migration and differentiation of epithelial cells [30,31]. The CK3 protein expression was in accordance with the gene expression analysis. CK3 protein expression indicates that epithelial cells had differentiated during wounding and maintains the corneal epithelial cell phenotype [32]. A recent study showed an increase in the in vitro migration rate of human corneal epithelial cells cultured in EGF-enriched media showed elevated expression of CK3 gene and protein [33].

Following injury, fibronectin was expressed abundantly and rapidly in the wounded region which marked the initiation of corneal epithelial wound healing mechanism [4]. Fibronectin acts as temporary extracellular matrix for the attachment of the migrating corneal epithelial cells towards the wounded region [34]. Fibronectin was expressed within one to eight hours after injury by fibroblasts and basal cells adjacent to the wounded region [34-36]. This study revealed that the fibronectin gene was expressed abundantly during initial day of wound creation especially in AH-supplemented media. Other studies also reported the increase expression of fibronectin in cornea after photorefractive keratectomy (PRK) [37,38] and mechanical abrasion injury [39]. Increase in the fibronectin expression is regulated by modulators namely cyclic adenosine 3’,-5’phosphate (cAMP), glucocorticoids, and growth factors such as EGF, TGF, and PDGF [4] which explained the increase in the expression of fibronectin in CCM group in the present study. Protein fibronectin reduced progressively during re-epithelisation [35,40] and three weeks after PRK as evidenced in immunohistology staining [37]. These results were in accordance to our study which showed reduction in fibronectin expression as the re-epithelialisation occurred.

CD44 gene expression is increased during injury to human eyes and mouse lens indicating its significance during inflammatory phase during wound healing [41,42]. Transcription of CD44 gene increased encircling wound margin three hours following epithelium injury and was the highest at 18 hours in the basal epithelial cells corresponding to the initiation of active migration [4]. Our findings revealed that AH supplemented in culture medium promoted migration during initial phase of wound healing. A significant up-regulation in the level of total CD44 mRNA at day 3 compared to initial day of wound creation, particularly in BM supplemented with AH. This is consistent with the hypothesis that the expression of CD44 offers adhesive strength for the epithelial sheet and cell-substratum interactions which mediates cell migration during corneal re-epithelialisation [4]. It has also been proposed that CD44 gene expression declines when epithelial cells proliferate and differentiated to restore the multi-layered epithelium [4]. In this study, re-epithelialisation through cell proliferation and differentiation was greater in the CCM supplemented with AH group compared to that of BM supplemented with AH group at day 3 causing reciprocal reduction of CD 44 gene expression. A reduction in CD44 gene expression in CEC cultured in AH-supplemented media at day 6 further suggests AH stimulated cell differentiation during re-epithelialisation. An increase in immunodeposition of CD44 protein has been reported after four hours at wound region in vivo alkali burn corneal injury, but reduced towards normal level after 7 weeks following complete wound closure and re-epithelisation [43].

Admittedly, there was a limitation in the study. A group comprising mixture of glucose and fructose equivalent to AH may have served as a good control. This would have further strengthened the healing effects of AH on the in vitro corneal abrasion wound healing model.

## Conclusion

The results of the present study showed Acacia honey (AH) accelerates wound closure of cultured CEC of the in vitro corneal abrasion wound healing model which were evident via migration study, morphology, gene and protein expression analyses. Acacia honey is a potential candidate for the establishment of Acacia honey based pharmaceutical eye drop for the treatment of corneal abrasion in the near future.
